# Diagnostic performance of the platelet-to-lymphocyte ratio (PLR) for periprosthetic joint infection after total hip and knee arthroplasty: a systematic review and meta-analysis

**DOI:** 10.1186/s43019-026-00314-8

**Published:** 2026-07-22

**Authors:** Alireza Sadeghpour Teymourlou, Amin Moradi, Hossein Akbari Aghdam, Hooman Yahyazadeh, Mohamm Ali Jafari Zare, Asghar Elmi, Mohammad Reza Aslani

**Affiliations:** 1 Department of Orthopedics, School of Medicine, Shohada Medical Research & Training Hospital, Tabriz University of Medical Sciences, Tabriz, Iran; 2 Department of Orthopedic Surgery, School of Medicine, Kashani Hospital, Isfahan University of Medical Sciences, Isfahan, Iran; 3 Department of Orthopedics, Bone and Joint Reconstruction Research Center, School of Medicine, Iran University of Medical Sciences, Tehran, Iran; 4 Department of Orthopedic Surgery, Faculty of Medicine, Tehran Medical Sciences Branch, Farhikhtegan Hospital, Islamic Azad University, Tehran, Iran; 5 Department of Physiology, Ardabil University of Medical Sciences, Ardabil, Iran; 6 Department of Orthopedics, Ardabil University of Medical Sciences, Ardabil, Iran

**Keywords:** Biomarkers, Meta-analysis, Platelet-to-lymphocyte ratio, Periprosthetic joint infection, Total joint arthroplasty

## Abstract

**Background:**

Research has investigated the function of biomarkers, such as the platelet–lymphocyte ratio (PLR), in identifying periprosthetic joint infection (PJI) after total hip arthroplasty (THA) and total knee arthroplasty (TKA). This study aimed to conduct an updated systematic review and meta-analysis in order to assess the diagnostic efficacy of PLR for PJI.

**Methods:**

Reviews of databases including PubMed, Scopus, and Web of Science were conducted to identify studies related to the use of PLR in diagnosing PJI. The evaluations covered the diagnostic performance of PLR for PJI. Furthermore, because of the significant heterogeneity across the studies, subgroup analyses were conducted focusing on geographic region (China versus other countries), site surgery (unseparated hip and knee versus separated hip and knee), and patient numbers (more than 200 versus less than 200).

**Results:**

A total of 4892 TKA and THA patients from 17 studies were included in the meta-analysis. Of the total patients, 1980 (40.5%) were diagnosed with PJI. Diagnostic sensitivity and specificity combined were 0.70 (95% CI 0.62–0.77) and 0.73 (95% CI 0.65–0.79), respectively. Results from the subgroup analysis indicated enhanced sensitivity and specificity concerning geographic differences, surgical site variances, and studies with fewer than 200 patients.

**Conclusions:**

PLR demonstrated moderate diagnostic accuracy and could function as an accessible supplementary marker in the evaluation process of PJI; additional research is necessary to establish optimal thresholds and its benefit alongside current diagnostic standards.

**Supplementary Information:**

The online version contains supplementary material available at 10.1186/s43019-026-00314-8.

## Introduction

Diagnosing periprosthetic joint infection (PJI) poses a significant challenge for surgeons performing arthroplasties [1]. Culture remains the primary method for diagnosing PJI, despite its limitations [2]. Various diagnostic methods have been employed to investigate pathogens present in blood or synovial fluid associated with the onset of PJI, and all have been met with controversy [3]. The assessment of PJI has included diagnostic measures such as C-reactive protein (CRP), erythrocyte sedimentation rate (ESR), as well as synovial fluid immune cell count [4]. Routine blood tests to evaluate inflammatory processes are often helpful for the early detection of PJI. Specifically, a complete blood count is simple to perform, cost-effective, and provides data on various cell types and morphological features, including the counts of white blood cells, lymphocytes, neutrophils, monocytes, platelets (PLT), and the average platelet volume [5]. Moreover, combined ratios derived from these measurements are also utilized as indicators of inflammation and have been proposed as biomarkers to assist in diagnosing, monitoring progression, and assessing risk in PJI [6]. The platelet-to-lymphocyte ratio (PLR), calculated as the platelet count divided by the lymphocyte count, has thus been suggested as a potential marker for PJI [7].

Platelets are recognized not only for their homeostatic duties but also for their active role against invading microorganisms [8]. Mean platelet volume alterations have been discovered in the presence of inflammatory conditions [9]. The acute phase reaction that occurs in inflammatory states can lead to a rise in platelets, functioning as part of the innate immune defense [10]. Extensive studies have indicated the connection between platelets and inflammatory responses in inflammatory conditions. The activation of platelets, their attachment to endothelial cells, and the release of inflammatory substances increase monocyte movement and adhesion to inflammation sites [11]. Inflammation can be worsened in cases such as PJI owing to platelet activation, which releases proinflammatory cytokines and chemokines [12], indicating the potential advantage of tracking platelet levels for patient care.

Moreover, it has been demonstrated that neutrophils and lymphocytes function as mediators and dynamic participants in inflammation [13]. Neutrophil elevation corresponds with inflammation, yet declining lymphocytes have been linked to increased inflammation susceptibility [14]. Therefore, applying biomarker ratios such as the platelet-to-lymphocyte ratio (PLR), which reflects an increase in platelet counts and a decrease in lymphocyte counts, could be useful in managing patients undergoing total hip arthroplasty (THA) and total knee arthroplasty (TKA). Although recent studies have focused on the use of the PLR biomarker to identify PJI [15], the role of PLR in the diagnosis of PJI has been ambiguous owing to the existence of distinct thresholds in studies, resulting in an ambiguous overall diagnostic efficacy. However, a number of systematic reviews and meta-analyses that compiled these studies faced notable limitations, including small samples and insufficient stratification (infection chronicity, reference standard, joint site), and rarely translated accuracy into clinical probabilities [16]. Therefore, a more comprehensive meta-analysis seems necessary. The goal of the present systematic review and meta-analysis was to examine how accurately PLR diagnoses PJI. In addition, another aim of this study was to characterize and explain heterogeneity using predefined subgroups and meta-analytic methods, and to contextualize clinical utility through pre- and post-test probabilities. Any residual heterogeneity is transparently addressed in the limitations section.

## Materials and methods

### Search strategy

This systematic review adhered to the Preferred Reporting Items for Systematic reviews and Meta-Analyses (PRISMA) guidelines for systematic reviews and meta-analyses, covering data up until October 2025. The study aimed to evaluate the diagnostic efficacy of PLR in the diagnosis of PJI in patients undergoing THA and TKA. Searching was undertaken using electronic databases such as Web of Science, PubMed, and Scopus. The search terms used were: (“PLR” OR “platelet-to-lymphocyte ratio” OR “platelet -lymphocyte ratio”) AND (“PJI” OR “periprosthetic infection” OR “periprosthetic joint infection”) AND (“diagnosis” OR “detection”). A manual search was also conducted on the basis of the reference lists of the selected articles. The search was conducted without restrictions on publication date, and only English-language articles were considered in the study. Studies examining the accuracy of PLR diagnosis in PJI cases were reviewed using retrospective, prospective, and clinical trial study designs.

### Eligibility criteria

The systematic review and meta-analysis criteria for inclusion involved the use of PLR in diagnosing PJI; adherence to reference standards such as those set by the Infectious Diseases Society of America (IDSA), Musculoskeletal Infections Society (MSIS), European Bone and Joint Infections Society (EBJIS), and International Consensus Meeting (ICM); establishment of distinct threshold values or test parameters for each study; and sufficient data to produce a 2 × 2 table.

Participants included in the study had undergone revision THA or TKA; had complete preoperative PLR data available; met the PJI diagnosis criteria outlined by MSIS, ICM, EBJIS, or IDSA guidelines; and had no restriction on the timeframe for diagnosing PJI as either chronic or acute.

Participants were excluded for the following reasons: (1) they had not undergone a revision arthroplasty; (2) no preoperative PLR data was available; (3) they suffered from inflammatory diseases such as rheumatoid arthritis; (4) were from case reports, conference abstracts, and review articles; and (5) the research involved joints other than the hip and knee (e.g., shoulder, elbow, ankle arthroplasty).

### Study assessment and data extraction

The titles and abstracts of the articles were first assessed by two independent reviewers, followed by a full-text evaluation. In the event of a disagreement over the review of the articles, the final assessment was conducted by the senior reviewer. Information gathered from each study consisted of the following: year of publication, authors, age, sex, criteria used for diagnosing PJI, study type (retrospective or prospective), type of arthroplasty employed, sensitivity values, specificity requirements, established cut-off points, positive likelihood ratio (positive LR), negative likelihood ratio (negative LR), and diagnostic odds ratio (DOR).

### Quality assessment

The quality of the study was assessed using the Quality Assessment of Diagnostic Accuracy Studies (QUADAS) framework.

### Statistical analysis

Statistical analyses were performed with the aid of STATA version 17 software and Meta-DiSC version 1.4. Calculations for true positive (TP), false positive (FP), false negative (FN), and true negative (TN) values were made for every study using the sensitivity, specificity, positive likelihood ratio, negative likelihood ratio, diagnostic score, and diagnostic odds ratio (DOR) metrics from qualified studies [17]. A bivariate diagnostic model accounting for random effects was utilized to evaluate the sensitivity and specificity of PLR. The findings were summarized and heterogeneity was analyzed by using forest plots derived from a random-effects framework. The *I*^*2*^ statistic was used to assess the variance among studies, with values below 50% implying minimal heterogeneity and those above 50% suggesting significant heterogeneity. The area under the curve (AUC) was calculated to summarize the overall diagnostic performance of PLR. The analysis identified a significance criterion of *P* < 0.05. The sensitivity analysis for each study was carried out using the “Midas” command within the STATA program. The changes in diagnostic effectiveness of PLR for PJI were quantified using Fagan plots, while Deek’s funnel plots analyzed publication bias. Publication bias tests were deemed statistically significant if their observed *P*-value was less than 0.05.

## Results

### Study selection

**Fig. 1:**
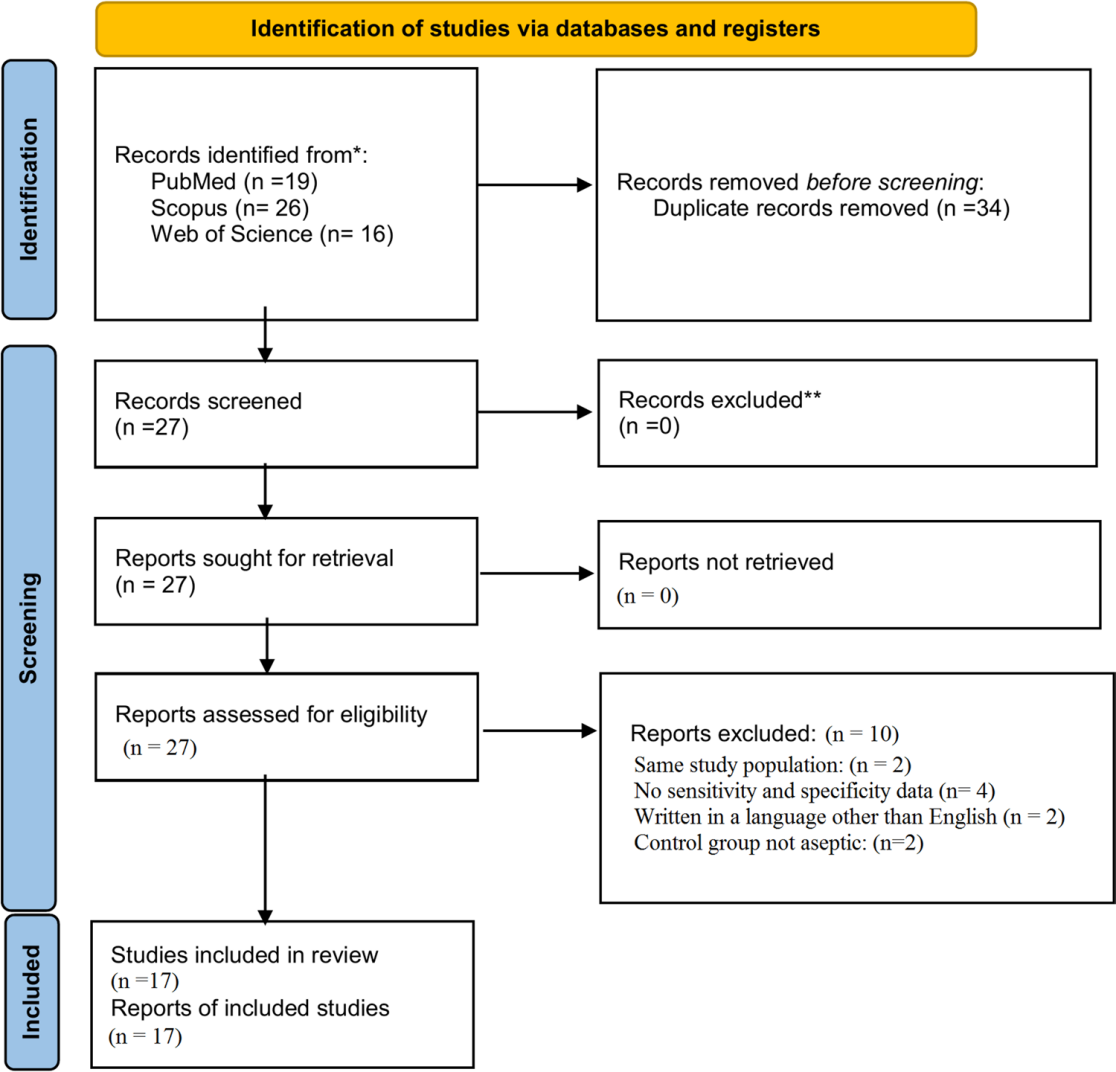
Flow diagram for new systematic reviews, which includes searches of database

A comprehensive search through the databases revealed 61 articles: 19 from PubMed, 26 from Scopus, and 16 from the Web of Science. Following the removal of 34 identified duplicates, 27 articles remained. A thorough examination of the article texts led to the decision to excluded ten studies. A total of 17 articles were eligible for inclusion in the study [5, 7, 12, 18–31]. However, Denye et al. [22] examined the results of PLR in two independent THA and TKA groups. Accordingly, 17 relevant articles yielding 18 results were chosen for inclusion in the systematic review and meta-analysis (Fig. 1).

### Quality assessment

Two authors carried out the quality assessment of all studies with the QUADAS-2 tool. Within the area of patient selection, a particular study was found to be of “high risk.” However, the quality assessment in the index test and reference standard categories rated the included studies as either “low risk” or “unclear.” Most of the studies included in this meta-analysis were assessed as being at low risk of bias (Supplementary Table 2).

### Study characteristics

This study comprised 4892 participants. A total of 1980 individuals, which accounted for 40.5%, were found to have a confirmed PJI. This review consists of research papers published within 2020 and 2025, each written entirely in English. One study was prospectively designed and 16 studies were retrospectively designed. In Europe, three studies were conducted, while four were carried out in the USA, and ten in Asia. Six studies utilized reference standards with ICM, seven studies with MSIS, two studies with IDSA, and two studies with EBJIS. Two studies did not specify the gender of six patients, leading to a total count of 2799 (57.3%) females. The number of patients undergoing THA/TKA surgery was not mentioned in two studies; however, in the remaining studies, there were 2169 (51.1%) TKA patients and 2076 (48.9%) THA patients. Findings on PLR for diagnosing PJI were drawn from ten studies that combined THA and TKA patient outcomes, with five focusing solely on TKA, and another three concentrating on THA. Across eight studies, PJI diagnosis was identified as chronic; one reported it as acute, four contained both acute and chronic cases (mixed), and four did not specify the timing. Table 1 gives a detailed summary of the characteristics for all studies. Table 2 presents the results obtained from data extraction across all studies, structured in a 2 × 2 format.

**Table 1:** Characteristics of the studies in meta-analysis for the diagnosis of PJI applying PLR

S/N	Study (year)	Country	Number of patients	Number of non-PJI/PJI	Gender (M/F)	Mean age (years)	Study design	Cut-off	Sample part	Type of infection	Sample collection	Ref. standard
1	Balato (2023) [18]	Italy	261	115/146	93/168	67.7	R	2.46	Knee: 261	chronic	NA	ICM criteria
2	Choe (2023) [19]	Japan	96	45/51	30/66	70	R	2.1	Knee: 96	chronic	2 months before surgery	ICM criteria
3	Deng (2024) [20]	China	168	110/58	71/97	67	R	3.79	Hip: 103 Knee: 65	Mixed	Admission day	MSIS criteria
4	Denyer (2023a) [21]	USA	577	330/247	281/293 (3 NA)	–	R	4.77	Hip: 291 Knee: 286	Chronic	< 4 weeks from index surgery	IDSA criteria
5	Denyer (2023b) [22]	USA	286	162/124	126/157 (3 NA)	–	R	3.62	Knee: 286	Chronic	< 4 weeks from index surgery	IDSA criteria
		USA	289	167/122	154/135	–	R	3.46	Hip: 289	Chronic	< 4 weeks from index surgery	IDSA criteria
6	Klemt (2023) [23]	USA	464	273/191	229/235	65	R	3.46	Hip: 464	Chronic	< 1 weeks from index surgery	EBJIS criteria
7	Kürüm (2024) [30]	Turkey	187	168/19	49/138	70	R	3.1	Knee: 187	Chronic	< 2 weeks from index surgery	ICM criteria
8	Maimait (2022) [7]	China	246	121/125	100/146	62	R	2.41	Hip: 147 Knee: 99	Chronic	Admission day	MSIS criteria
9	Moldovan (2024) [24]	Romania	77	45/32	41/36	63	R	2.63	Hip: 77	NA	1 day before surgery	EBJIS criteria
10	Shi (2023) [25]	China	244	157/87	130/114	65	R	2.18	Hip: 166 Knee: 78	Mixed	Admission day	ICM criteria
11	Song (2024) [26]	China	158	79/79	66/92	66	R	181.51	Hip: 90 Knee: 68	NA	Second day of admission	MSIS
12	Tirumala (2021) [12]	USA	538	332/206	268/270	66	R	3.62	Knee: 538	Chronic	Preoperatively	MSIS criteria
13	Wang (2021) [31]	China	221	139/82	87/134	64	R	2.36	Hip: 141 Knee: 80	Mixed	7 days before surgery	MSIS criteria
14	Wu (2022) [27]	China	164	117/47	60/104	66	R	2.71	Hip: 115 Knee: 49	Mixed	First or second day of admission	MSIS criteria
15	Xu (2022) [5]	China	543	298/245	246/297	62	R	2.9	Hip: – Knee: –	NA	Admission day	ICM criteria
16	Yu (2025) [28]	China	269	176/93	136/133	65	P	2.73	Hip: 193 Knee: 76	NA	Admission day	ICM criteria
17	Zhao (2020) [29]	China	104	78/26	54/50	63	R	2.77	Hip: – Knee: –	Acute	Preoperatively	MSIS criteria

EBJIS, European Bone and Joint Infections Society; F, female; ICM, International Consensus Meeting; IDSA, Diseases Society of America; M, male; MSIS, Musculoskeletal Infections Society; NA, not applicable; P, prospective study; PJI, periprosthetic joint infection; R, retrospective study; Ref. standard, reference standard

**Table 2:** Data extracted for the construction of a 2 × 2 table

S/N	Study (year)	TP	FP	FN	TN	Total
1	Balato (2023)	113	59	33	56	261
2	Choe (2023)	42	18	9	27	96
3	Deng (2024)	37	30	21	80	168
4	Denyer (2023a)	144	79	103	251	577
5	Denyer (2023b) knee	77	21	47	141	286
	Denyer (2023b) hip	98	98	24	69	289
6	Klemt (2023)	145	47	46	226	464
7	Kürüm (2024)	19	92	0	76	187
8	Maimait (2022)	71	20	54	101	246
9	Moldovan (2024)	24	14	8	31	77
10	Shi (2023)	26	20	61	137	244
11	Song (2024)	43	12	36	67	158
12	Tirumala (2021)	161	58	45	274	538
13	Wang (2021)	42	27	40	112	221
14	Wu (2022)	40	57	7	60	164
15	Xu (2022b)	177	128	68	170	543
16	Yu (2025)	54	48	39	128	269
17	Zhao (2020)	23	10	3	68	104

TP, true positive; FP, false positive; FN, false negative; TN, true negative

### PLR diagnostic accuracy

**Fig. 2:**
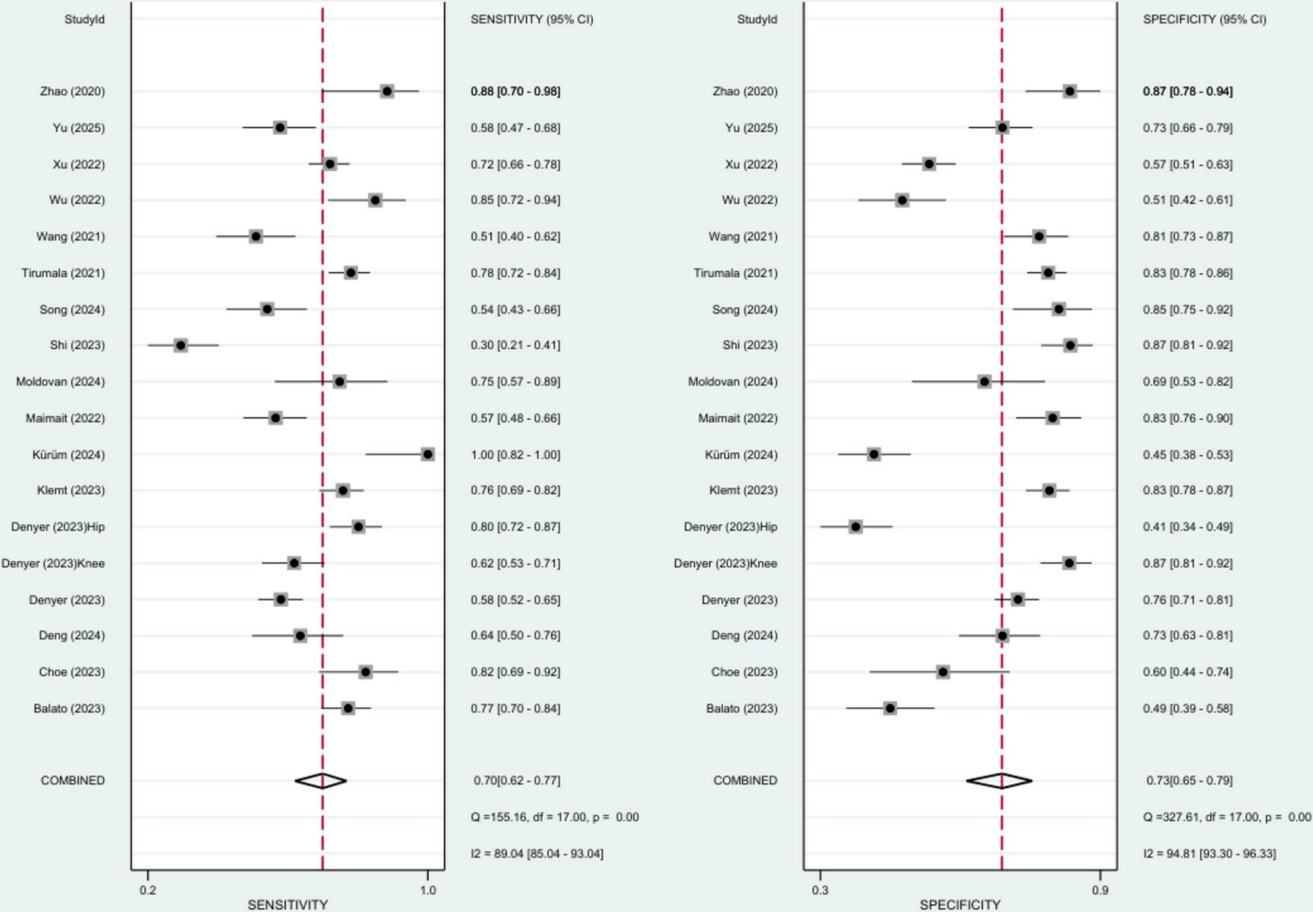
Forest plots of sensitivity and specificity

**Fig. 3:**
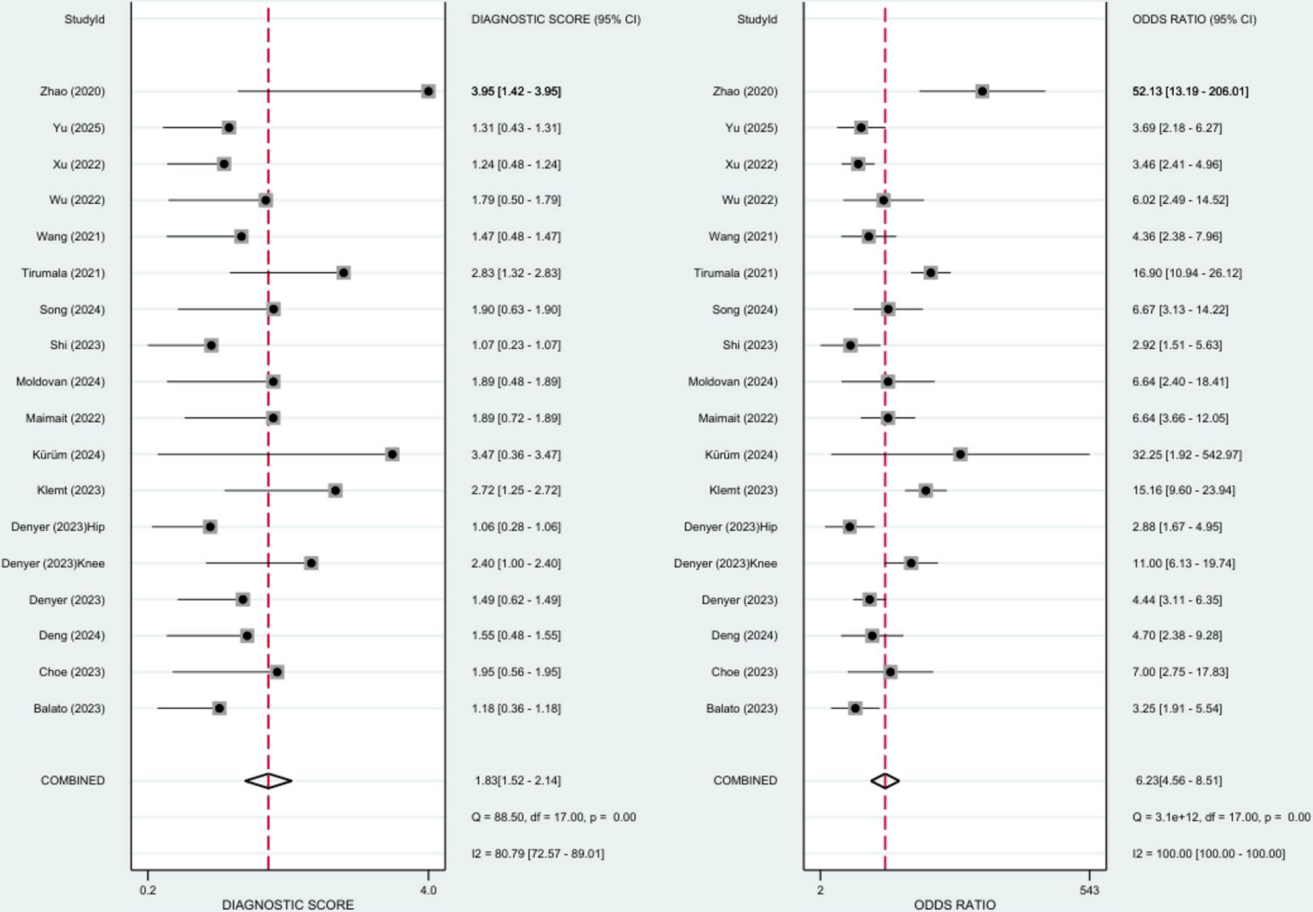
Diagnostic odd-ratio (DOR) and diagnostic score

**Fig. 4:**
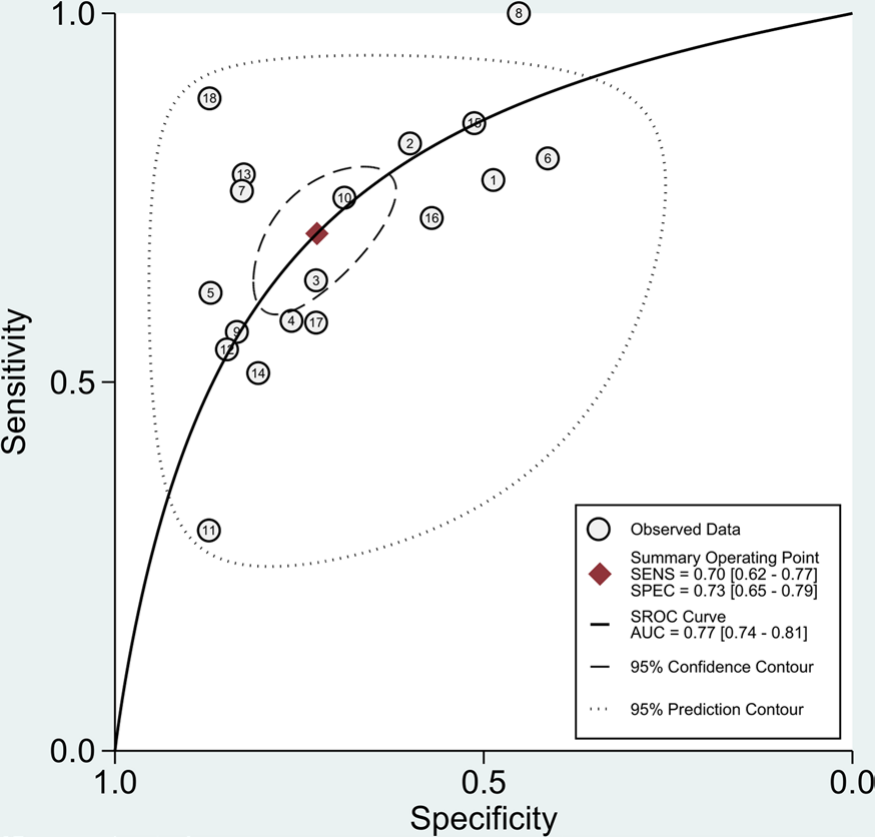
Summarized receiver operating characteristic (SROC) curve of sequencing-based diagnosis performance

Diagnostic sensitivity and specificity combined were 0.70 (95% CI 0.62–0.77) and 0.73 (95% CI 0.65–0.79), respectively (Fig. 2), but marked heterogeneity was present in the studies, which was indicated by *I*^*2*^ values of 89% (95% CI 85–93%) and 94.8% (95% CI 93.3–96.3%). The total DOR and diagnostic score respectively came out to be 6.23 (95% CI 4.56–8.51) and 1.83 (95% CI 1.52–2.14) (Fig. 3). The AUC value for PLR for PJI cases is at 0.77, with a 95% confidence interval of 0.74–0.81 (Fig. 4).

### Clinical application evaluation

**Fig. 5:**
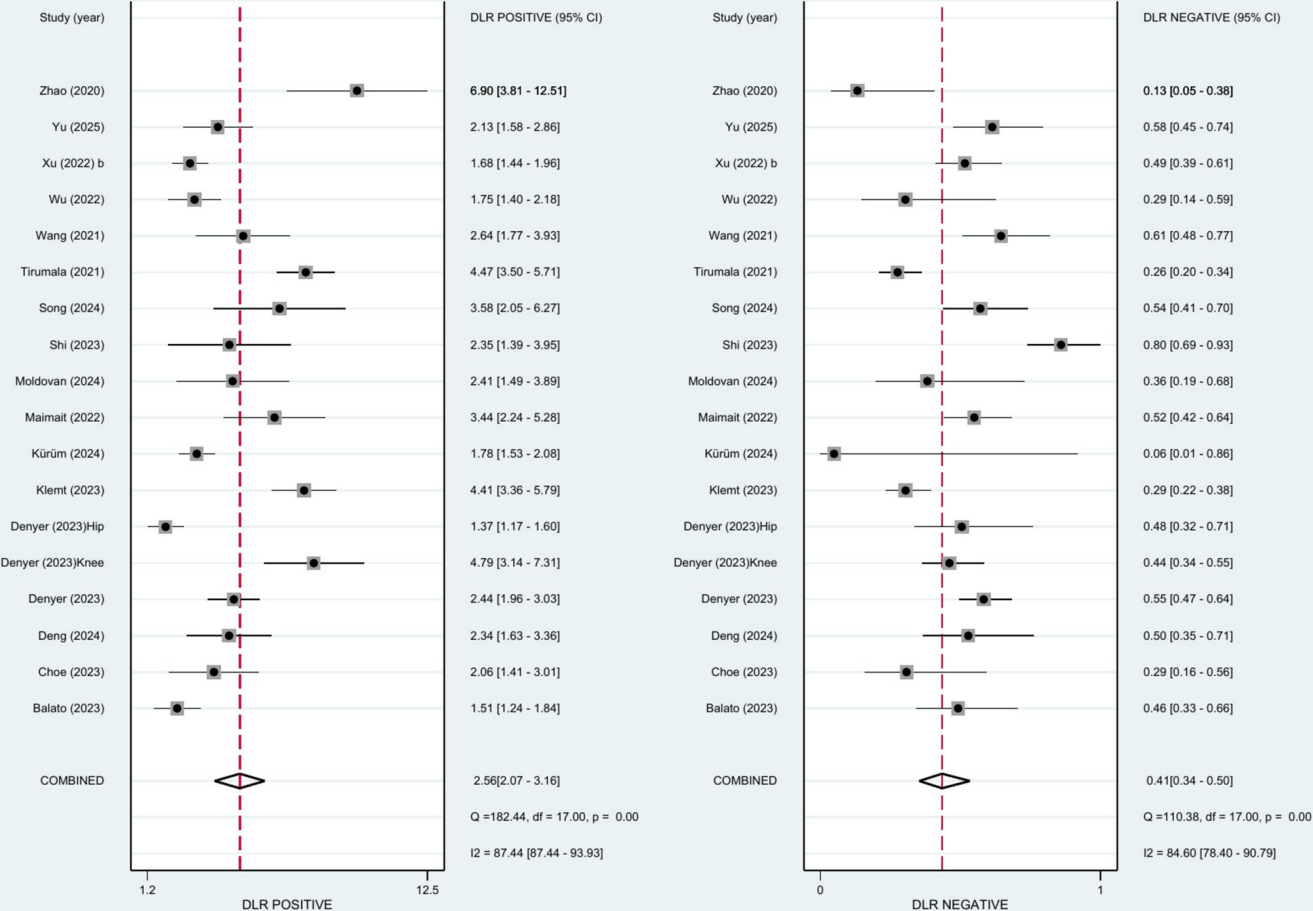
Forest plots of pooled likelihood ratio (positive likelihood ratio and negative likelihood ratio)

**Fig. 6:**
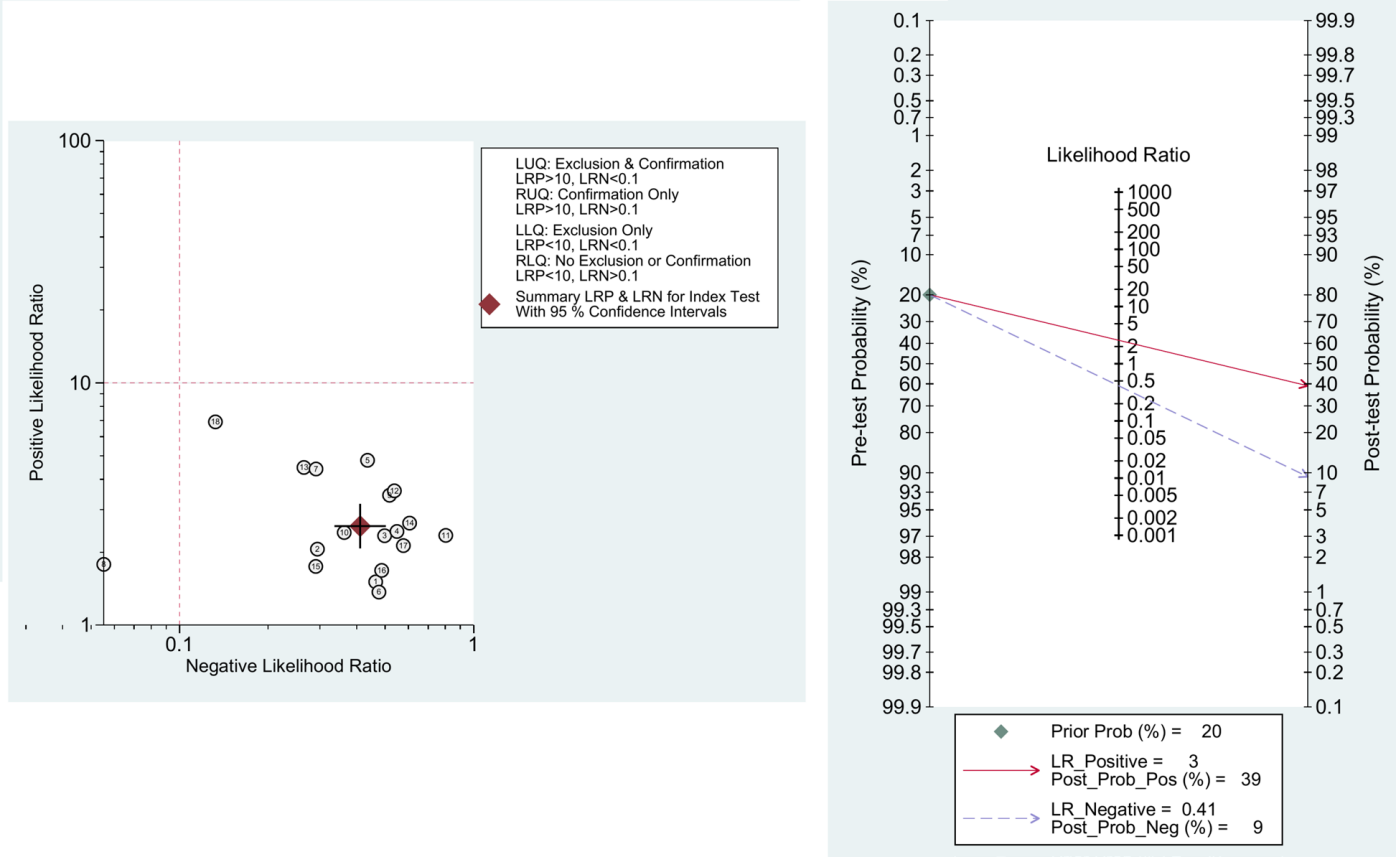
Pooled likelihood ratio scatter diagrams (**A**) and Fagan’s nomogram (**B**) of platelet-to-lymphocyte ratio value for diagnosis of PJI

The combined positive likelihood ratio and negative likelihood ratio for diagnosing PJI were 2.56 (95% CI 2.07–3.16) and 0.41 (95% CI 0.34–0.50), respectively (Fig. 5). On the basis of previous studies, the 20% pre-test probabilities were used for calculation of the post-test probability owing to the relatively low incidence of PJI [32]. Consequently, 0.2 pre-test probabilities were employed to estimate the post-test probability by integrating the likelihood ratio with the initial probability. The pre-test and post-test probabilities of PLR for diagnosing PJI clinically were assessed using Fagan’s nomogram. According to the Fagan nomogram, the upper slash line indicated a positive likelihood ratio of 3, with a pre-test probability of 20% and a resulting post-test probability of 39%. The pre-test probability was established at 20%, with the lower slash having a negative likelihood ratio of 0.41, which resulted in a post-test probability of 9% (Fig. 6A). Figure 6B displays outcomes suggesting that the combined effect of PLR in diagnosing PJI lies in the lower right quadrant, meaning + LR is less than 10 and −LR is greater than 0.1, indicating that section has neither diagnostic nor exclusional capabilities.

### Heterogeneity analysis

#### Meta-regression

**Fig. 7:**
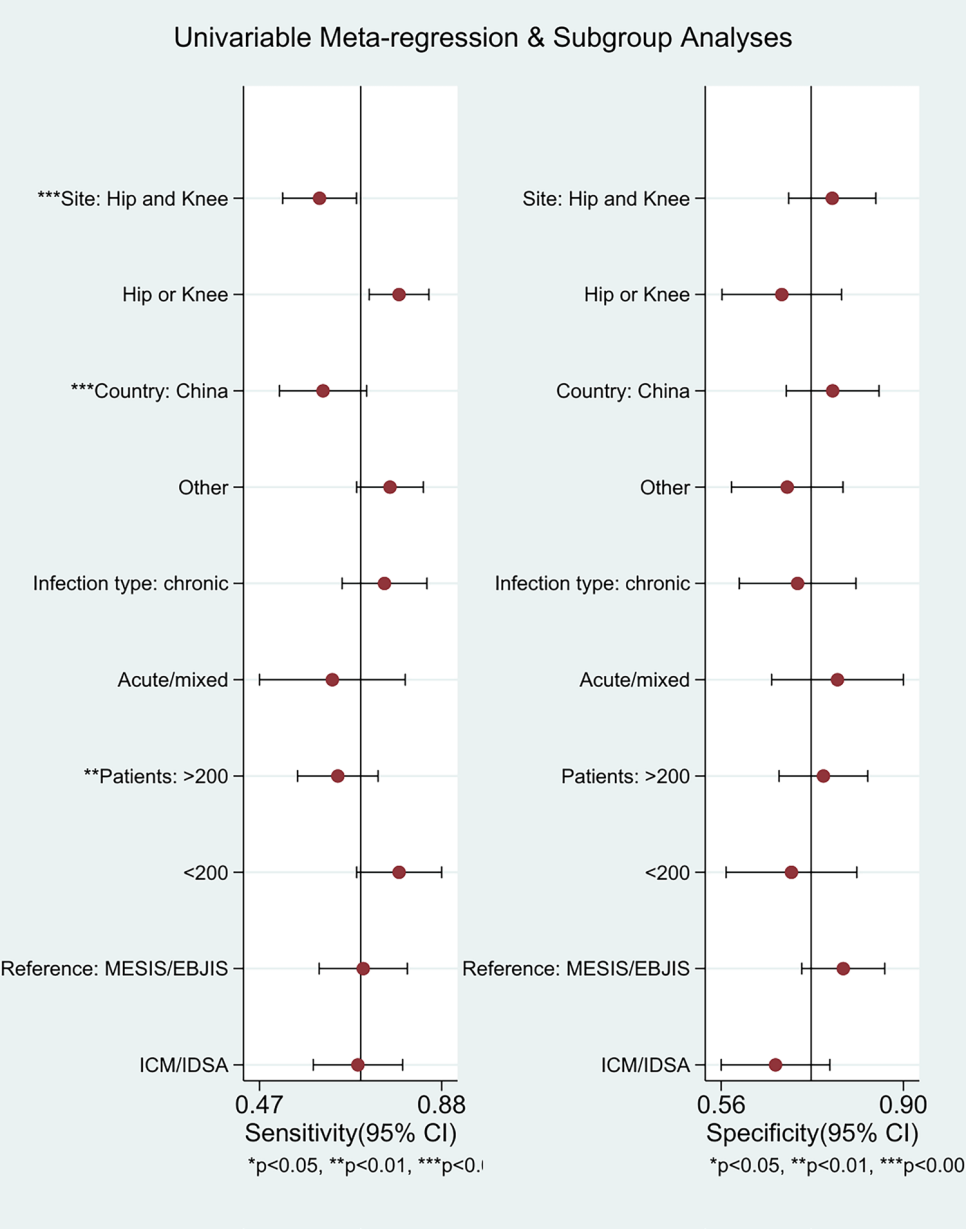
Meta-regression analysis for platelet-to-lymphocyte ratio with several variables

A meta-regression analysis was performed to identify the causes of variation (Fig. 7). The sensitivity and specificity results were associated with the varying numbers of patients (> 200 versus < 200), diverse reference standards (MSIS/EBJIS versus ICM/IDSA), site surgery (unseparated hip and knee versus separated hip and knee), diagnosis time of PJI (chronic versus other), and geographical locations of the countries where the studies were conducted (China versus other countries). To delve deeper into the diverse sources of variation, an analysis of subgroups was performed.

#### Subgroup analysis

The pooled sensitivity and specificity for subgroups with fewer than 200 samples were 0.79 (95% CI 0.69–0.88) and 0.69 (95% CI 0.57–0.81), respectively, whereas for subgroups with more than 200 samples, these values were 0.65 (95% CI 0.56–0.74) and 0.75 (95% CI 0.67–0.83), respectively. With a *P*-value of 0.00 for sensitivity and 0.29 for specificity, the *I*^*2*^ value was recorded at 49% (Table 3).

**Table 3:** Subgroup analysis of PLR values for PJI diagnosis

Subgroup analyses	No. of studies	No. of patients	Estimates (95% CI)				
			Sensitivity	*P*-value	Specificity	*P*-value	*I*^*2*^ (%)
Overall studies	17 study with 18 results	4892	0.70 (0.62–0.77)	–	0.72 (0.65–0.79)	–	–
Sample site(s)							
Hip or knee Hip; knee (both)	8 10	2144 2748	0.79 (0.72–0.86) 0.61 (0.52–0.69)	0.00	0.67 (0.56–0.78) 0.77 (0.68–0.85)	0.39	75
Number of patients							
< 200 > 200	7 11	954 3938	0.79 (0.69–0.88) 0.65 (0.56–0.74)	0.00	0.69 (0.57–0.81) 0.75 (0.67–0.83)	0.29	49
Reference standard							
MSIS or EBJIS ICM or IDSA	9 9	2194 2698	0.71 (0.61–0.81) 0.70 (0.59–0.80)	0.13	0.79 (0.71–0.86) 0.66 (0.56–0.76)	0.49	70
Country							
China Other	9 9	2171 2772	0.62 (0.52–0.72) 0.77 (0.69–0.84)	0.00	0.77 (0.68–0.85) 0.68 (0.58–0.79)	0.32	60
Infection type							
Acute/mixed Chronic NA	5 9 4	901 2944 1047	0.64 (0.47–0.80) 0.76 (0.66–0.85)	0.90	0.78 (0.65–0.90) 0.70 (0.59–0.81)	0.06	97

Values for the sensitivity and specificity of the pooled reference standard were 0.71 (95% CI 0.61–0.81) and 0.79 (95% CI 0.71–0.86) for the MSIS/EBJIS, and 0.70 (95% CI 0.59–0.80) and 0.66 (95% CI 0.56–0.76) for the ICM/IDSA criteria. The *P*-value was 0.13 for sensitivity, 0.49 for specificity, and the *I*^*2*^ value reached 70% (Table 3).

In the geographical location subgroup for countries, the pooled sensitivity and specificity values were 0.62 (95% CI 0.52–0.72) and 0.77 (95% CI 0.68–0.85) for China, while these values were 0.77 (95% CI 0.69–0.84) and 0.68 (95% CI 0.58–0.79) for other countries. The *P*-value for sensitivity stood at 0.00, while for specificity it was recorded as 0.32, with an *I*^*2*^ value of 60% (Table 3).

Values for the sensitivity and specificity of the pooled site surgery were 0.61 (95% CI 0.52–0.69) and 0.77 (95% CI 0.68–0.85) for the unseparated hip and knee location, and 0.79 (95% CI 0.72–0.86) and 0.67 (95% CI 0.56–0.78) for the separated hip and knee location. Sensitivity showed a *P*-value of 0.00; specificity had a *P*-value of 0.39, with an *I*^*2*^ value measured at 75% (Table 3).

Values for the sensitivity and specificity of the pooled PJI diagnostic time were 0.76 (95% CI 0.66–0.85) and 0.70 (95% CI 0.59–0.81) for chronic, and 0.64 (95% CI 0.47–0.80) and 0.78 (95% CI 0.65–0.90) for the acute/mixed studies. With a sensitivity *P*-value of 0.90 and a specificity *P*-value of 0.06; the *I*^*2*^ statistic stood at 97% (Table 3).

### Sensitivity analysis and publication bias

**Fig. 8:**
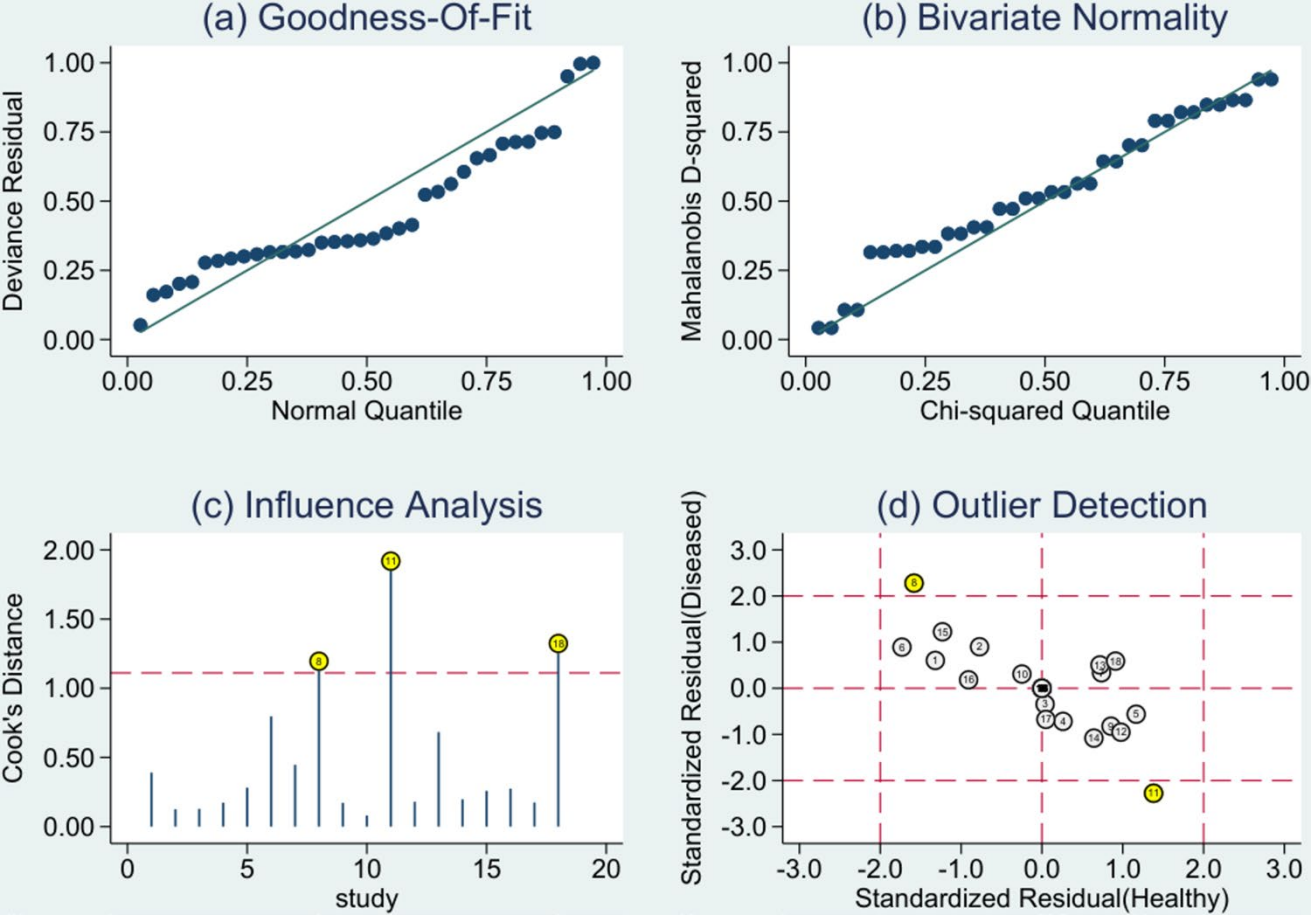
Sensitivity analysis of platelet to lymphocyte ratio in the diagnosis of PJI. (**A**) goodness-of-fit. (**B**) Bivariate normality. (**C**) Influence analysis. (**D**) Outlier detection

**Fig. 9:**
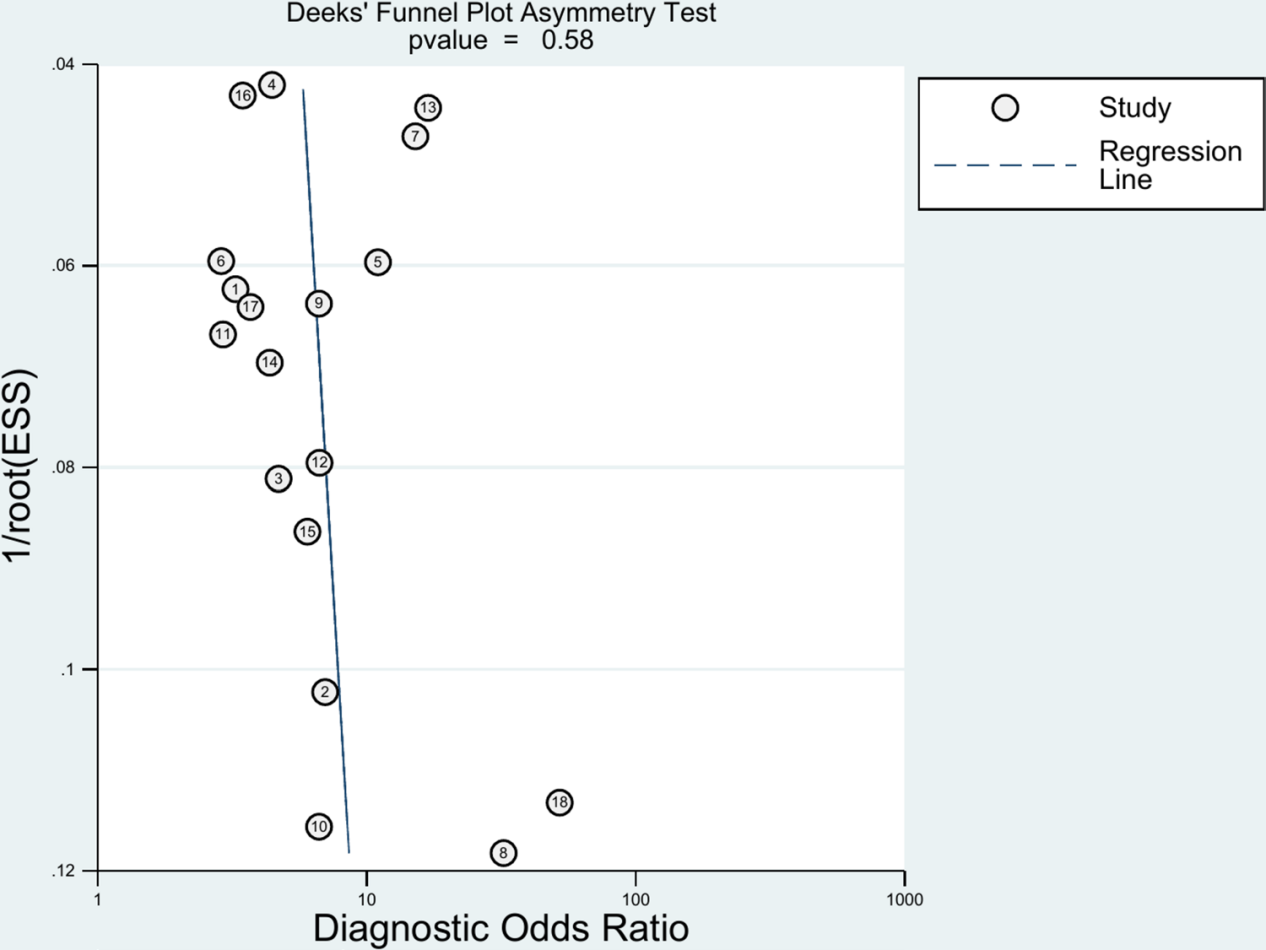
Funnel plot for publication bias assessment of included studies

Analyzing sensitivity findings revealed that both the goodness-of-fit and bivariate normality supported the suitability of the random-effects bivariate model for the study (Fig. 8A, B). According to the impact analysis, three studies were found to have a large weight (Fig. 8C). Analyzing the outliers indicated that two specific studies could contribute to the heterogeneity (Fig. 8D). With the outlier data excluded from the study, the AUC was determined to be 0.77 (95% CI 0.73–0.81). The data showed no variation, pointing to a good degree of stability within the study. No significant asymmetry in Deek’s funnel plot was found for DOR (*P* = 0.58), suggesting a possible absence of publication bias (Fig. 9).

## Discussion

A meta-analysis of 17 articles involving a total of 4892 patients. The combined diagnostic accuracy of the PLR index was 0.70 (95% CI 0.62–0.77) for sensitivity and 0.73 (95% CI 0.65–0.79) for specificity. The pooled DOR for PLR was also 6.23, with a range of 4.56–8.51. Assessments of both positive and negative likelihood ratios, combined with post-test probability data from moderate clinical settings, revealed a medium-level diagnostic utility as a complementary measure for diagnosing PJI. By conducting additional subgroup analysis, it was found that sampling fewer than 200 patients, using samples solely from knee or hip cases, and focusing on countries besides China led to enhanced sensitivity/specificity. Interestingly, subgroup analysis revealed a noticeable decrease in the studies’ heterogeneity.

One of the most significant complications arising from knee and hip replacement surgery is PJI [33]. Studies have demonstrated that between 1% and 2% of patients undergoing TKA and THA procedures are at risk of developing PJI, necessitating prolonged antibiotic therapy [34]. Currently, no single widely accepted method exists for diagnosing PJI despite the availability of multiple established reference points. Research into studies such as neutrophil-to-lymphocyte ratio (NLR), PLR, and monocyte-to-lymphocyte ratio (MLR) is heavily focused on biomarkers owing to their affordability and ease of access [35]. The use of biomarkers is constrained by their limited ability to distinguish between different conditions. High concentrations of biomarkers can be substantially increased in numerous diseases or following trauma, thereby impacting the diagnostic precision of PJI [36].

Neutrophils contribute to worsening inflammation via inflammatory mediators, unlike lymphocytes, which act to moderate the inflammatory reaction [37]. Elevated neutrophil counts and reduced lymphocyte levels in inflammatory conditions have been demonstrated to modify immune responses. In addition, the involvement of platelets in inflammation has been clearly established. Platelets have a dual function in managing inflammation and regulating immune responses. On one side, platelets are crucial for hemostasis and wound healing, as they employ defense strategies such as moving pathogens across their plasma and internal membranes and demonstrating antimicrobial capabilities [11, 38]. However, the interplay between infectious agents and platelets can propagate infection by inducing disseminated intravascular coagulation (DIC) [39], elevating pro-inflammatory and anti-inflammatory cytokine network activity [40], releasing adhesion molecules (such as intercellular adhesion molecule 1 [ICAM1] and vascular cell adhesion protein 1 [VCAM1]) [41], providing a communicative link between innate and adaptive immunity [42], carrying a number of chemokines and cytokines [38], and causing endothelial cell damage through the release of reactive oxygen species [11].

The inflammatory response activates platelets, prompting interaction with leukocytes. By releasing vascular endothelial growth factor (VEGF) and platelet-derived growth factor (PDGF), platelets play a role in the inflammatory response, facilitating leukocyte migration and infiltration [43]. The use of platelet-related biomarkers, including platelet count, PLR, and the platelet count to mean platelet volume ratio, in diagnosing various metabolic, prothrombotic disorders, neoplastic conditions, and PJI has been evaluated recently [9, 12, 44, 45].

The current meta-analysis study examined the relationship between PLR and the diagnosis of PJI in patients undergoing THA and TKA. Several meta-analyses examined the relationship between PLR and the diagnosis of PJI, including research by Festa et al. [16]. The study’s results comprised five studies that had combined sensitivity and specificity of around 0.77 and 0.75, respectively. Furthermore, the considerable heterogeneity in their research findings (48.89% for sensitivity and 95.34% for specificity) indicates that additional studies are required. However, systematic reviews and meta-analyses that have evaluated other biomarkers for diagnosing PJI have reported varying results. For example, neutrophil-to-lymphocyte ratio (NLR) showed a sensitivity of 0.73 and specificity of 0.75 [46], monocyte-to-lymphocyte ratio (MLR) demonstrated a sensitivity range of 0.54–0.81 and a specificity between 0.78 and 0.81 [16], and platelet count/mean platelet volume ratio (PVR) exhibited a sensitivity of 0.72 and specificity of 0.77 [16]. However, most of these studies faced limitations such as high heterogeneity and a limited number of included studies, making it difficult to draw definitive conclusions about the efficacy of each biomarker.

Some methodological and demographical factors have been demonstrated to influence the outcomes of diagnostic studies. Geographical location, patient age, PJI diagnosis timing, design of study, reference standards, surgical site, patient enrollment numbers, sampling methods, antibiotic application, and existing comorbidities influence biomarker diagnostic outcomes [16, 47]. Therefore, conducting subgroup analyses according to methodological parameters may prove beneficial when evaluating heterogeneity of studies for future study design. The present study found that a subgroup analysis of the findings according to sample site was a factor that enhanced the sensitivity/specificity of the research, with studies that focused only on THA or TKA individually displaying a sensitivity of 0.79, contrasting with a sensitivity of 0.61 for research that evaluated both sites. These findings highlight the significance of selecting patients when investigating the diagnostic role of PLR in the diagnosis of PJI. Further investigation is needed to understand the potential differences caused by varying blood supply levels or the size of the surgical area.

Geographical location differences were another factor that increased the sensitivity of studies on the basis of subgroup analysis. Studies carried out in China yielded a sensitivity rate of 0.62, which contrasted with a sensitivity rate of 0.77 in other countries. The subgroup analysis by geographical location showed that although heterogeneity decreased, it remained present among the studies. These results indicate possible differences related to geography, ethnicity, or the methods used for sample collection and processing. Therefore, geographical factors should be considered when determining an accurate cutoff value for the diagnostic accuracy of PLR in diagnosing PJI.

Another factor contributing to the increased sensitivity of studies was the number of patients, with studies involving fewer than 200 patients having a sensitivity of 0.79 compared with 0.65 for studies with a sample size exceeding 200 patients. In addition, subgroup analysis based on patient numbers showed that a significant reduction in study heterogeneity was observed. The diversity of patients in a large sample size is likely to impact the sensitivity and specificity of PLR for diagnosing PJI owing to differences in inclusion and exclusion criteria, various clinical conditions, and comorbidities, necessitating further research. Notably, in subgroup analysis based on reference standards, a specificity of 0.71 was observed for studies utilizing MSIS or EBJIS criteria, whereas it was 0.70 for studies using ICM or IDSA. Although the sensitivity and specificity improved according to the subgroup analysis of reference standards, it was clear that the heterogeneity among the studies remained unchanged. These findings suggest that the increased sensitivity and specificity observed in the subgroup analysis may stem from variations in how different criteria are defined—such as those relevant to acute versus chronic infection, differences in the quantitative thresholds for CRP or ESR, etc.—highlighting the need for further research. The findings imply that criteria involving measured variables with likely differences should be utilized with caution when assessing the diagnostic power of PLR for PJI.

## Limitations

These limitations can be encapsulated in the following points. The main limitation of the research was the high degree of variability among the studies, despite the fact that a random-effects model was utilized in the meta-analysis. Although subgroup analysis was performed to examine heterogeneity of studies in the current study, another limitation of the study was the high heterogeneity that remained in the current study. Therefore, it seems that appropriate design of PLR diagnostic studies for PJI seems methodologically essential in future studies. The included studies were conducted in a retrospective manner, which can be seen as a limitation. A limitation was the uncertainty surrounding the application of antibiotics across various studies, potentially leading to a rise in false-negative results. Despite performing subgroup analyses related to when PJI was diagnosed in this study, examining PLR findings for PJI with regard to its chronic or acute nature could not be done accurately because of data constraints.

## Conclusions

The present meta-analysis findings indicated that PLR has a moderate diagnostic value for PJI in patients undergoing revision THA and TKA. Given the moderate sensitivity and specificity alongside modest likelihood ratios, it is more fitting to consider PLR as an accessible supplementary marker with moderate diagnostic precision, rather than a completely adequate diagnostic tool on its own. Owing to the diversity of the studies, additional research is suggested to establish further validation on a larger scale to identify the most effective PLR threshold values.

## Supplementary Information

Supplementary Material 1.

## Data Availability

The datasets used and/or analyzed during the current study are available upon reasonable request from the corresponding author.
